# Immunological Hypoglycemia Associated with Insulin Antibodies Induced by Exogenous Insulin in 11 Chinese Patients with Diabetes

**DOI:** 10.1155/2015/746271

**Published:** 2015-04-15

**Authors:** Heng Quan, Huiwen Tan, Qianrui Li, Jianwei Li, Sheyu Li

**Affiliations:** Department of Endocrinology and Metabolism, West China Hospital of Sichuan University, Chengdu, Sichuan 610041, China

## Abstract

*Aims*. To investigate the characteristics of immunological hypoglycemia associated with insulin antibodies (IAbs) induced by exogenous insulin in Chinese patients with diabetes. *Methods*. The clinical data of patients with immunological hypoglycemia due to IAbs were retrospectively analyzed by screening patients with diabetes discharged from West China Hospital from 2007 to 2013. *Results*. A total of 11 patients (eight men and three women) were identified. Insulin-C-peptide separation was found in all patients via insulin and C-peptide release test. Previous insulin use was ceased after admission and was switched to oral hypoglycemic agents (OHAs) (8/11), lifestyle modification only (2/11), or regular human insulin (1/11). Hypoglycemia was ameliorated after a median of 20 days (interquartile range [IQR], 11–40), while IAbs turned negative after a median of 17 months (IQR, 4–19), and serum immunoreactive insulin (IRI) levels dropped substantially after a median of 22 months (IQR, 9–32) in these cases. *Conclusions*. In insulin-treated patients with unexpected and refractory hypoglycemia even after insulin therapy was gradually reduced or even withdrawn, IAbs induced by exogenous insulin should be considered, and insulin withdrawal might be promptly needed. The course of immunological hypoglycemia was benign and self-limited.

## 1. Introduction

Hypoglycemia is one of the most important complications of diabetes. It is commonly observed in diabetic patients on insulin therapy and also occurs occasionally after the cessation of insulin. Immunological hypoglycemia associated with insulin antibodies (IAbs) induced by exogenous insulin was first reported by Harwood in 1960 [1]. It is a relatively rare but important subtype of unexpected hypoglycemia in insulin-treated patients with diabetes even after they have stopped insulin administration. It has been poorly described, usually ignored, and difficult to manage. Little experience has been summarized before.

In the present study, we tended to retrospectively analyze the clinical characteristics of a cohort of patients with hypoglycemia associated with IAbs induced by exogenous insulin in a tertiary referral center, to help clinicians better understand the manifestation and management of such condition.

## 2. Subjects and Methods

### 2.1. Study Design and Patients

We retrospectively reviewed 6,824 patients with diabetes discharged from the Department of Endocrinology and Metabolism in West China Hospital of Sichuan University from January 2007 to December 2013.

Patients were eligible for inclusion in the study if they had hypoglycemia as one of the discharge diagnoses, received insulin therapy prior to admission with positive IAb, and had available results from blood glucose monitoring, standard meal tolerance test (MTT), and insulin and C-peptide release test during hospitalization. Patients were excluded if they had self-reported ever use of methimazole, tiopronin, glutathione, or other thiol-containing agents or had other established causes of hypoglycemia, such as excessive use of oral hypoglycemic agents (OHAs) or insulin.

Additionally, we selected patients hospitalized during the same period with negative IAb and no record of unexpected hypoglycemia episodes, matched to each case patient by insulin type, age (within a 3-year range), and gender in a 1 : 1 ratio, as controls to evaluate the effects of the agents' cross-reaction in the insulin test.

Our study was carried out in accordance with the Declaration of Helsinki and Good Clinical Practice guidelines.

### 2.2. Data Collection

The demographic profiles, clinical characteristics, and laboratory data of all subjects were collected. All laboratory tests were conducted in the Department of Laboratory Medicine in West China Hospital of Sichuan University. Standard MTT and insulin and C-peptide release test began at 08:00 a.m. after a 10-hour overnight fast. Blood samples were drawn for glucose, insulin, and C-peptide determination before and 2 hours after a loading meal containing 75 g carbohydrates without insulin usage in the morning. Plasma glucose was measured by hexokinase method (Roche, Germany). Levels of serum immunoreactive insulin (IRI) and C-peptide reactivity (CPR) were determined by electrochemiluminescence immunoassay (ECLIA) method (Roche, Germany). Serum alanine transaminase (ALT) and aspartate transaminase (AST) were measured by the method without pyridoxal phosphate activation suggested by the International Federation of Clinical Chemistry (IFCC) (Roche, Germany). Serum creatinine was measured by the Jaffe method (Roche, Germany). Estimated glomerular filtration rate (eGFR) was calculated by the Chronic Kidney Disease Epidemiology Collaboration (CKD-EPI) equation. Insulin antibody (IAb), islet cell antibody (ICA), and glutamic acid decarboxylase antibody (GAD-Ab) were evaluated with enzyme-linked immunosorbent assay (ELISA) method (Biomerica, CA, USA). Each sample was run in duplicate, and optical density (OD) was measured in an ELISA reader at 405 nm. An index was calculated based on the average of the results of each sample for IAb and ICA: Index = (OD of test sample)/(OD of quality control reference). The “positive IAb” or “positive ICA” was defined as an index > 1.05 (test sample A_405 nm_/quality control reference A_405 nm_ > 1.05). The “negative IAb” or “negative ICA” was defined as an index < 0.95 (test sample A_405 nm_/quality control reference A_405 nm_ < 0.95). The “positive GAD-Ab” and “negative GAD-Ab” were defined as an average value of GAD-Ab > 1.05 U/mL and <1.00 U/mL, respectively. If the value stayed critical (0.95–1.05 for IAb and ICA, 1.00–1.05 U/mL for GAD-Ab), we repeated the test or drew new parallel sample and tested again. IAb, ICA, and GAD-Ab of the patients were recorded as positive or negative without exact value. Blood glucose was monitored by ACCU-CHEK Active nine times per day (preprandially, 2 h postprandially, 10:00 p.m., 0:00 a.m., and 3:00 a.m.) for all patients since admission, and if hypoglycemic symptoms occurred at any other time, blood glucose would also be monitored immediately. Type of hypoglycemia, namely, preprandial or postprandial (defined as 2 hours from meal), and at night (defined as between 10:00 p.m. and 06:00 a.m.), was classified as the time range when more than half of hypoglycemic episodes occurred. Unexpected hypoglycemia was identified as refractory hypoglycemic episodes occurring 3 days after the cessation of insulin.

### 2.3. Statistical Analysis

Continuous variables including age, body mass index (BMI), course of diabetes, duration of insulin treatment, ALT, AST, creatinine, eGFR, glycated hemoglobin (HbA1c), and the lowest blood glucose were presented as medians and interquartile ranges (IQR) due to an inadequate sample size and uncertain distribution. Categorical variables including episodes of hypoglycemia and insulin usage were presented as frequencies. Analyses were performed with Origin software, version 8.0 (OriginLab) and Statistical Package for Social Sciences 20.0 (SPSS, Chicago, IL). Paired chi-square test was used in comparing dichotomous variables between groups, when Wilcoxon signed rank test was used in comparing continuous data. All statistical tests were two-tailed and *P* < 0.05 was considered statistically significant.

## 3. Results

A total of 11 patients with unexpected hypoglycemia (eight men and three women) were eventually identified with a median age of 70 years (IQR, 61–77). The median course of diabetes was 48 months (IQR, 12–120). The median serum ALT, AST, and eGFR were 26 IU/L (IQR, 15–38), 24 IU/L (IQR, 15–35), and 88.07 mL/min/1.73 m^2^ (IQR, 74.48–99.64), respectively (Table 1). Compared with patients without unexpected hypoglycemia, patients with unexpected hypoglycemia had a smaller BMI (21.00 kg/m^2^ [IQR, 19.90–21.64] versus 25.15 kg/m^2^ [IQR, 20.06–29.07], *P* = 0.036), a trend of shorter duration of insulin treatment without statistical significance (5 months [IQR, 4–12] versus 36 months [IQR, 24–60], *P* = 0.050), lower insulin dosage (15 IU [IQR, 14–20] versus 26 IU [IQR, 24–30], *P* = 0.016), lower fasting PG (5.93 mmol/L [IQR, 4.52–6.30] versus 8.89 mmol/L [IQR, 7.19–12.17], *P* = 0.041), lower HbA1c (6.4% [IQR, 5.9–7.3] versus 8.3% [IQR, 6.7–11.8], 46 mmol/mol [IQR, 41–51] versus 67 mmol/mol [IQR, 50–105], *P* = 0.041), higher fasting and after MTT 2 h IRI (hi pmol/L [IQR, 2437.20-hi] versus 69.12 pmol/L [IQR, 29.82–93.42], *P* = 0.003; hi pmol/L [IQR, hi-hi] versus 187.62 pmol/L [IQR, 113.88–339.36], *P* = 0.003; hi was defined as insulin > 6000.00 pmol/L) and CPR (1.810 nmol/L [IQR, 1.010–3.130] versus 0.768 nmol/L [IQR, 0.462–0.905], *P* = 0.010; 3.350 nmol/L [IQR, 3.100–4.220] versus 1.470 nmol/L [IQR, 1.350–1.840], *P* = 0.003), and higher frequency of hypoglycemia (3 episodes/week [IQR, 1–5] versus 1 episode/week [IQR, 0-1], *P* = 0.007) (Table 3).

All the patients received insulin therapy for blood glucose control prior to admission, and the insulin strategies of each patient were shown in Table 1. The median dose of insulin in patients with unexpected hypoglycemia was 15 IU (IQR, 14–16) per day. Seven patients received premixed insulin, while the other four received rapid acting insulin. As shown in Table 1, after a median time of 5 months (IQR, 4–12) from the initiation of insulin therapy, unexpected hypoglycemia occurred recurrently in these patients even after discontinued insulin therapy. Six patients (cases 4–7, 10, and 11) had more frequent hypoglycemia during night than during day-time, and the frequent day-time hypoglycemia of the other five patients was usually preprandial. The median lowest blood glucose monitored was 2.0 mmol/L (IQR, 1.3–2.7). Four patients (cases 3, 6, 7, and 11) experienced disorders of consciousness such as confusion or coma. And one patient (case 7) receiving Humalog Mix25 also experienced insulin allergy presented as rashes, redness, and itches at the injection site. For all patients with unexpected hypoglycemia, insulin overdose was the initially suspected cause of hypoglycemia. Thus the dose of insulin was gradually reduced in all these patients or even discontinued in six patients (cases 2, 3, 5, 7, 8, and 11) by patients themselves or by physicians prior to admission. However, spontaneous hypoglycemia occurred recurrently even after discontinuation of insulin, and thus they were admitted to the hospital for further examination and management.

Their laboratory tests after admission indicated positive IAb in all patients with unexpected hypoglycemia, positive ICA in case 6, and positive GAD-Ab in case 10. The fasting and 2 h IRI concentrations after MTT significantly increased in these patients, while the C-peptide levels were relatively low, which was described as insulin-C-peptide separation (Table 2). However, insulin-C-peptide separation was not observed in patients without unexpected hypoglycemia and their IRI concentrations were much lower (Table 3), which indicated a low cross-reaction of the test reagent with exogenous insulin. Therefore, the increased serum IRI in patients with unexpected hypoglycemia could be considered endogenous and the hypoglycemic attacks might be due to IAb-insulin dissociation.

To relieve hypoglycemia, the previously used insulin was reduced and then withdrawn in all patients who developed unexpected hypoglycemia after admission, with replacement of other blood glucose control strategies. Cases 3, 4, 5, 8, and 9 received *α*-glucosidase inhibitor 3 times per day instead, case 1 received glinide twice per day (before breakfast and lunch), case 11 received *α*-glucosidase inhibitor in combination with biguanide, and case 6 received triple drugs, namely, glinide, *α*-glucosidase inhibitor, and dipeptidyl peptidase-IV (DPP-IV) inhibitor. Case 10 switched to regular recombinant human insulin from premixed insulin. Cases 2 and 7 only received diet control and advice to exercise after previous insulin withdrawal. Moreover, case 7, who experienced both hypoglycemia and allergy, received glucocorticoid therapy. Methylprednisolone was infused intravenously 40 mg/d for 3 days, followed by oral prednisone at 30 mg/d. Prednisone was reduced gradually and discontinued in 16 days, when the allergic skin reaction was alleviated as well.

After cessation of insulin, hypoglycemia was still observed recurrently during hospitalization (Table 1), although it improved generally in these patients in the present study. The median time when hypoglycemic episodes vanished during hospitalization after previous insulin withdrawal was 20 days (IQR, 11–40).

Seven patients with unexpected hypoglycemia were followed up after discharge. Six of them (cases 2–5, 8, and 9) got complete remission of hypoglycemia, while case 1 had a significant reduction of the frequency of hypoglycemia (only two episodes before breakfast during the 9-month follow-up). IAbs turned negative in four cases (cases 3, 4, 8, and 9) after a median of 17 months (IQR, 4–19). HbA1c was 44.5 mmol/mol (IQR, 42–50) or 6.25% (IQR, 6.0–6.7) in six cases (cases 2–5, 8, and 9) after a median of 23.5 months (IQR, 15–31). Standard MTTs and insulin and C-peptide release tests were conducted during follow-up in six cases (cases 1–3, 5, 8, and 9), and serum IRI levels substantially dropped after a median of 22 months (IQR, 9–32) (Table 2).

## 4. Discussion 

Hypoglycemia is a common problem in diabetic patients with antidiabetic therapies, especially in those receiving insulin treatment. It is highly associated with mortality and cardiovascular events as illustrated by a number of studies [2–5]. Most episodes of hypoglycemia occurred due to insulin overdose or irregular food intake and could be easily prevented by insulin dose adjustment or lifestyle modification. However, hypoglycemia due to other causes could also present refractory after modification of insulin dose and food intake. In the current study, patients presented with unexpected hypoglycemia even after discontinuation of insulin and with obvious insulin-C-peptide separation in insulin and C-peptide release test. Thus, insulin autoimmune hypoglycemia due to IAbs should be considered, and etiological treatment should be given to diminish the episodes [6].

As established already, IAbs could be induced by methimazole and other thiol-containing agents [7, 8], as well as exogenous insulin injection [9, 10]. Methimazole-induced IAb is highly associated with unexpected hypoglycemia and insulin resistance and could be difficult to treat, requiring glucocorticoid and other immunosuppressive treatments in most cases [11, 12]. IAb induced by exogenous insulin is common in insulin-treated patients, especially in those years when human insulin was not derived and used in clinical practice [13–15]. However, it is not always associated with hypoglycemia or insulin resistance, and IAb-associated hypoglycemia is usually reported as single cases in the literature [16–18].

To our knowledge, the present report is the largest case series of IAb-associated immunological hypoglycemia in China, indicating that IAb is of clinical significance and should be considered in patients with persistent and unexplained hypoglycemia even after insulin withdrawal, especially in old male with long-term use of rapid insulin analogues or neutral protamine Hagedorn (NPH).

The present study (Table 1) showed that insulin analogues and NPH could be associated with IAbs production in insulin-treated patients with diabetes. Insulin analogues, which differ from human insulin, have been proved to cause IAbs response [15, 19, 20]. NPH, a heterologous protein, could be antigenic itself and is also an adjuvant to increase the antigenicity of exogenous insulin [21, 22].

The accuracy of insulin measurement affected the results. In the present study, insulin concentration was measured by the ECLIA method with a monoclonal antibody, which reported a cross-reactivity of <0.02% for insulin aspart and insulin lispro [23, 24], 19.2% for porcine insulin, and 0.05% for proinsulin (PI) (provided by the instruction of kit). Additionally, blood samples in all patients were drawn for IRI determination without insulin usage in the morning to reduce cross-reaction. Furthermore, patients without unexpected hypoglycemia were recorded with a larger insulin dosage and a lower IRI concentration than patients with unexpected hypoglycemia, suggesting that the extremely high levels of IRI in patients with unexpected hypoglycemia could not be explained by exogenous insulin. Consequently, the measured IRI in our study could be considered the true insulin level [25], which included IAb-binding insulin and free insulin [23]. Although the C-peptide test kit with a monoclonal antibody had a cross-reactivity of 32.5% for PI, PI concentrations in fasting status were 100 times less than C-peptide concentration (provided by the instruction of kit), and the measured CPR was the true C-peptide level as well [26].

The genetic basis of IAb-associated immunological hypoglycemia was not fully understood. Human leukocyte antigen (HLA) was reported to play a role and individuals with HLA-DR4 genotype were susceptible to develop IAb-associated immunological hypoglycemia [27, 28], resembling that of a patient with insulin autoimmune syndrome (IAS) [8, 29], type 1 diabetes mellitus [30, 31], or rheumatoid arthritis [32, 33]. However, HLA typing was not performed in this retrospective study.

To be clarified, two types of IAbs have been revealed by previous studies: high affinity with low capacity and low affinity with high capacity [34, 35]. And the IAbs in patients with immunological hypoglycemia induced by exogenous insulin has been found to be the latter type [28]. The circulating IAbs may act as an insulin binding reservoir, impacting the amount of biologically active insulin and leading to higher postprandial blood glucose levels after a subcutaneous insulin injection, but when a large portion of insulin dissociates from the IAbs, this process is independent of the plasma glucose level, leading to the onset of unexpected hypoglycemia [27, 36].

Our results also showed that IAbs could turn negative in most patients after insulin withdrawal given sufficient follow-up duration, which was consistent with a previous study [37] demonstrating that IAbs might disappear one year after insulin withdrawal. This indicated that IAb was not a life-long antibody, and immunological hypoglycemia was benign and self-limited. All our patients withdrew previous insulin to minimize antigen stimulation, which was of importance in the treatment of immunological hypoglycemia, and most of them received OHAs as replacement, if the residual *β*-cell function could supply the essential insulin demands of the patients. However, transient hypoglycemia should be alerted due to the release of previously combined insulin from the IAb-insulin complex when the autoantibody was erased. Generally, the hypoglycemia attacks relieved in parallel with the decrease in the titer of the IAb and serum insulin level after insulin therapy was discontinued.

As ICA and GAD-Ab were detected in cases 6 and 10, respectively, latent autoimmune diabetes in adults (LADA) could be suspected when taking into consideration their history of diabetes and their laboratory tests. Patients with LADA were insulin-free for at least six months after diagnosis because of tolerable postprandial C-peptide concentration and they could be managed by OHAs in this period of time [38]. Case 6 used glinide (starlix) to replace insulin administration and his glycemic control was improved upon discharge. However, he was lost to follow-up and thus later condition was unknown, although LADA patients using controversial agents, such as glinide, needed long-term observation. The present case suggested that clinicians should be cautious with the choice of OHAs for patients with LADA and with rigorous follow-up.

On the other hand, some patients would have to change insulin type if they had a low postprandial C-peptide concentration such as case 10 who was diagnosed with diabetes for six years. Of note, the changing of insulin type was proved to be effective. Recombinant human insulin, an ideal alternative, has least antigenicity but could still lead to IAbs production, either because of the fact that the insulin injected in a foreign site was immunogenic or because of the occurrence of subcutaneous polymerization [39–41]. Therefore, OHAs are better for non-insulin-dependent diabetic patients than insulin, because of its effects on minimizing the possibility of IAbs induced by exogenous insulin.

Glucocorticoid may be helpful in extremely serious cases or in patients hypersensitive to insulin. Glucocorticoid could work dramatically and was supposed to be withdrawn within weeks. Immunosuppressive agents or plasmapheresis could be another option for severe cases according to previous reports [28, 42].

IAbs could be considered a complication of the treatment with exogenous insulin. As a result, IAb-associated hypoglycemia should be considered when initiating insulin therapy. Most of the patients in our case series ceased insulin therapy and glycemic control remained satisfying, indicating that nonessential prescription of insulin was common in Chinese patients with diabetes. Proper use of exogenous insulin, especially insulin analogs, should be reconsidered by both diabetologists and general practitioners.

The present study has several limitations. Firstly, this is a single-centered retrospective observational and descriptive study with a limited number of patients and a limited duration of follow-up. Patients with positive IAb but without episodes of unexpected hypoglycemia were not recruited in this study. Secondly, information from telephone follow-up was occasionally used in the analysis of two cases (cases 2 and 5), which might lead to some recall biases. Thirdly, insulinoma-associated protein 2 (IA-2) antibody, the exact titer of IAb, and IAb affinity and capacity were not measured, and thus it was difficult to quantify the change of titers of IAbs in these patients. Therefore, further well-designed larger prospective cohort studies are still to be suggested with predefined endpoints of laboratory and clinical parameters, including IA-2 antibody, the exact titer of IAb, affinity and capacity of IAb, and HLA typing.

## 5. Conclusions 

In summary, hypoglycemia in insulin-treated patients with diabetes might result from various causes, among which immunological causes associated with IAbs shall not be neglected, although it is a rare etiology and insulin overdose should always be addressed before considering other causes. If unexpectedly recurrent hypoglycemia occurred, especially after insulin had been discontinued for several days, immunological hypoglycemia associated with IAbs due to exogenous insulin should be considered. Lab tests for insulin concentration, C-peptide concentration, and IAb titer could help make diagnosis. Metformin, *α*-glucosidase inhibitor, and DPP-IV inhibitors appeared to be alterative blood glucose control options for minimizing the risk of hypoglycemia.

## Figures and Tables

**Table 1:** General data of 11 cases with immunological hypoglycemia associated with IAbs.

Case	Sex	Age	BMI	Course of diabetes	Duration of insulin treatment	Serum ALT	Serum AST	Serum creatinine	eGFR	Insulin types	Frequency of insulin use	Insulin dosage	Type of hypoglycemia^*^	Frequency of hypoglycemia in first week during hospitalization
	M/F	Years	kg/m^2^	Months	Months	IU/L	IU/L	umol/L	mL/min/1.73 m^2^		Times/day	IU/day		Episodes/week
1	F	77	21.23	5	4	38	48	54.3	100.28	NovoMix 30	2	14	Before breakfast	5
2	F	55	18.73	36	5	8	14	64.3	88.34	NovoRapid	3^†^	12^†^	Before breakfast	1
3	M	46	21.88	8	7	15	21	89.9	88.07	NovoMix 30	2	22	Before lunch	3
4	F	77	25.54	48	21	13	15	83.0	58.80	NovoMix 30	2	16	At night	1
5	M	62	21.64	24	12	26	24	94.1	74.48	NovoRapid +Lantus	4	20^†^	At night	2
6	M	79	NA	120	108	15	15	112.0	58.30	NovoRapid	2	40	At night	1
7	M	70	19.90	12	1	27	38	89.4	77.50	Humalog Mix25	2	14	At night	10
8	M	71	20.34	204	4	32	26	73.7	96.57	Humalog Mix25	2	8	Before dinner	4
9	M	65	20.76	96	0.13	38	35	66.1	111.48	NovoRapid	3	14	Before breakfast	6
10	M	61	NA	72	4	40	34	74.4	99.64	Wan Sulin 30R^#^	NA	NA	At night	4
11	M	82	NA	180	12	26	16	82.0	76.44	Novolin 30R	NA	16	At night	2

M: male, F: female, BMI: body mass index, ALT: alanine transaminase, AST: aspartate transaminase, eGFR: estimated glomerular filtration rate, and NA: not available.

^*^Type of hypoglycemia, namely, preprandial or postprandial (defined as 2 hours from meal) and at night (defined as between 10:00 p.m. and 06:00 a.m.), was classified as the time range when more than half of hypoglycemic episodes occurred.

^†^These data were obtained via phone calls, which might lead to some recall biases.

^#^Wan Sulin 30R contained 30% porcine insulin and 70% protamine zinc porcine insulin, which was produced by Wanbang Biopharmaceuticals in China.

**Table 2:** Results of 11 cases with immunological hypoglycemia associated with IAbs on admission and during follow-up.

Case	On admission							During follow-up							Alternative treatment after admission
	Fasting status			After meal tolerance test 2 h			HbA1c	Fasting status			After meal tolerance test 2 h			HbA1c	
	PG mmol/L	Serum IRI^*^ pmol/L	Serum CPR nmol/L	PG mmol/L	Serum IRI^*^ pmol/L	Serum CPR nmol/L	% (mmol/mol)	PG mmol/L	Serum IRI^*^ pmol/L	Serum CPR nmol/L	PG mmol/L	Serum IRI^*^ pmol/L	Serum CPR nmol/L	% (mmol/mol)	
1	6.05	>6000.00	4.930	21.00	>6000.00	6.660	5.6 (38)	5.28	1075.20	1.140	20.46	2061.00	2.700	NA	NovoNorm 0.5 mg bid
2	3.66	>6000.00	2.530	16.66	>6000.00	3.160	7.3 (56)	5.92	103.68	0.512	15.86	343.80	2.750	6.1 (43)	Lifestyle modification
3	4.97	1697.40	1.010	13.24	>6000.00	3.800	6.1 (43)	6.84	36.66	0.681	5.90	126.90	2.140	6.0 (42)	Glucobay 50 mg tid
4	5.44	4342.20	0.675	12.65	>6000.00	4.220	5.7 (39)	NA	NA	NA	NA	NA	NA	6.4 (46)	Glucobay 50 mg tid
5	5.93	>6000.00	3.380	17.86	>6000.00	7.130	5.9 (41)	6.54	110.94	0.639	13.39	441.00	2.650	5.2 (33)	Glucobay 25 mg tid
6	9.08	>6000.00	3.130	26.09	>6000.00	3.320	9.6 (81)	NA	NA	NA	NA	NA	NA	NA	Starlix 60 mg tid, Glucobay 100 mg tid, Januvia 100 mg qd
7	4.52	2437.20	1.110	17.85	>6000.00	3.100	6.3 (45)	NA	NA	NA	NA	NA	NA	NA	Glucocorticoid^†^, lifestyle modification
8	8.37	4493.40	1.810	19.53	>6000.00	3.350	6.5 (48)	5.74	258.66	NA	12.96	445.44	NA	6.7 (50)	Glucobay 50 mg tid
9	3.86	>6000.00	1.050	17.38	>6000.00	1.970	6.4 (46)	7.90	1021.80	0.817	18.01	2241.60	1.950	7.1 (54)	Glucobay 75 mg tid
10	6.10	1185.60	0.580	18.80	2958.60	1.590	6.8 (51)	NA	NA	NA	NA	NA	NA	NA	Gansulin R^#^ 4IU tid
11	6.30	>6000.00	2.670	23.70	>6000.00	3.480	8.6 (70)	NA	NA	NA	NA	NA	NA	NA	Glucobay 50 mg tid, Glucophage 500 mg bid

PG: plasma glucose, IRI: immunoreactive insulin, CPR: C-peptide reactivity, and NA: not available.

^*^Insulin concentration conversion formula was described as*μ*U/mL × 6 = pmol/L (http://diabetes.diabetesjournals.org/site/misc/SIunits.pdf).

^†^Methylprednisolone was infused intravenously 40 mg/d for 3 days, followed by oral prednisone 30 mg/d, which was reduced gradually and discontinued on day 16.

^#^Gansulin R is biosynthetic human insulin produced by Dongbao Pharmaceutical Co., Ltd., in China.

**Table 3:** Characteristics of the patients with unexpected hypoglycemia and patients without unexpected hypoglycemia.

Variable	Patients with unexpected hypoglycemia (*n* = 11)	Patients without unexpected hypoglycemia (*n* = 11)	*P* value
Male (%)	8 (72.73)	8 (72.73)	1.000
Age, median (IQR), years	70 (61–77)	70 (60–77)	0.608
BMI, median (IQR), kg/m^2^	21.00 (19.90–21.64)	25.15 (20.06–29.07)	0.036
Course of diabetes, median (IQR), months	48 (12–120)	120 (84–156)	0.139
Duration of insulin treatment, median (IQR), months	5 (4–12)	36 (24–60)	0.050
Serum ALT, median (IQR), IU/L	26 (15–38)	23 (20–34)	0.722
Serum AST, median (IQR), IU/L	24 (15–35)	25 (23–30)	0.683
Serum creatinine, median (IQR), umol/L	82.0 (66.1–89.9)	88.0 (74.0–106.0)	0.328
eGFR, median (IQR), mL/min/1.73 m^2^	88.07 (74.48–99.64)	72.49 (51.05–93.96)	0.374
Insulin dosage, median (IQR), IU/day	15 (14–20)	26 (24–30)	0.016
Fasting status on admission			
PG, median (IQR), mmol/L	5.93 (4.52–6.30)	8.89 (7.19–12.17)	0.041
Serum IRI^*^, median (IQR), pmol/L	hi (2437.20–hi)^†^	69.12 (29.82–93.42)	0.003
Serum CPR, median (IQR), nmol/L	1.810 (1.010–3.130)	0.768 (0.462–0.905)	0.010
After meal tolerance test 2 h on admission			
PG, median (IQR), mmol/L	17.86 (16.66–21.00)	19.34 (16.20–21.14)	0.657
Serum IRI^*^, median (IQR), pmol/L	hi (hi–hi)^†^	187.62 (113.88–339.36)	0.003
Serum CPR, median (IQR), nmol/L	3.350 (3.100–4.220)	1.470 (1.350–1.840)	0.003
HbA1c			
median (IQR), %	6.4 (5.9–7.3)	8.3 (6.7–11.8)	0.041
median (IQR), mmol/mol	46 (41–51)	67 (50–105)	
Frequency of hypoglycemia in first week during hospitalization, median (IQR), episodes/week	3 (1–5)	1 (0-1)	0.007

BMI: body mass index, ALT: alanine transaminase, AST: aspartate transaminase, eGFR: estimated glomerular filtration rate, PG: plasma glucose, IRI: immunoreactive insulin, and CPR: C-peptide reactivity.

^*^Insulin concentration conversion formula was described as: *μ*U/mL × 6 = pmol/L (http://diabetes.diabetesjournals.org/site/misc/SIunits.pdf).

^†^hi was defined as insulin >6000.00 pmol/L.
